# Impact of the First Phase of the COVID-19 Pandemic on the Acquisition of Goods and Services in the Italian Health System

**DOI:** 10.3390/ijerph19042000

**Published:** 2022-02-11

**Authors:** Martina Capuzzo, Gian Luca Viganò, Cinzia Boniotti, Lucia Maria Ignoti, Claudia Duri, Veronica Cimolin

**Affiliations:** 1 ASST Spedali Civili di Brescia-UOC Ingegneria Clinica, 25123 Brescia, Italy; martina.capuzzo@asst-spedalicivili.it (M.C.); gianluca.vigano@asst-spedalicivili.it (G.L.V.); cinzia.boniotti@asst-spedalicivili.it (C.B.); lucia.ignoti@asst-spedalicivili.it (L.M.I.); claudia.duri@asst-spedalicivili.it (C.D.); 2 Department of Electronics, Information and Bioengineering, Politecnico di Milano, 20133 Milano, Italy

**Keywords:** COVID-19, procedure, purchasing, PPE, emergency management, crisis, supply chain management

## Abstract

The emergency caused by the escalation in the COVID-19 pandemic, which became widespread starting on 31 January 2020, put a strain on the Italian National Health System and forced purchasing centres to deviate from the ordinary general principles dictated by current legislation. The aim of this paper is to describe how Spedali Civili Hospital in Brescia challenged the crisis, structured itself optimally, followed simplified procedures, launched new processes, and opened up more Intensive Care Unit beds to accommodate the high number of COVID cases. From an analysis of the equipment variation in terms of increased purchases, subsequent installations, and tests carried out compared with the pre-pandemic period, we report the difficulties that hospitals had to face in the first phase of the pandemic and how they were able to respond to their needs. Our data clearly displayed how the pandemic situation led to a deep internal reorganisation and that the drafting of simpler, effective, and adaptable procedures represents a first key element to ensure receptivity and responsiveness in the management of ordinary and non-ordinary events such as this pandemic condition.

## 1. Introduction

From November 2019, COVID-19 began to spread, first in China and then worldwide. The acronym derives from the initials Co = corona; v = virus; id = infectious disease; and 19 = 2019, the year the disease began to spread. The causative agent of the disease is SARS-CoV2, which causes severe and acute respiratory syndrome [1]. The rapid spread of this epidemic led to a declaration of a global pandemic by the World Health Organization (WHO) on 11 March 2020 [2].

**Figure 1:**
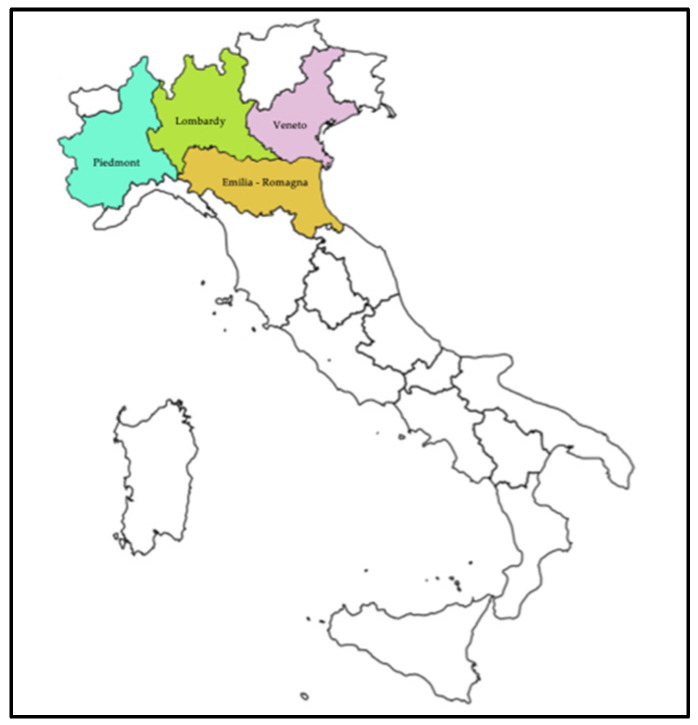
The most affected region during the first phase.

The first two coronavirus cases registered in Italy date to 29 January at the Lazzaro Spallanzani Institute in Rome, while the first case of secondary transmission was established in Codogno, a Lombard municipality in the province of Lodi, on 18 February 2020. The severity of the situation is well reflected by the following two figures (Table 1 and Table 2). In Table 1, a representation of the infection curve is reported for the most affected regions during the first phase (Figure 1). The numbers of Lombardy stand out, with more than 35,000 cases on 26 April. The region also proved to be the most affected and strained, even in terms of institutional and health response.

In May 2020, Italy had a total of 232,664 infections and 33,340 deceased, with 88,758 and 16,079, respectively, attributable to Lombardy alone, which in terms of percentage of deceased, was 48.23% of the national total (Table 2).

The number of infections increased rapidly, and the Italian Health System had to face and deal with an emergency of extraordinary magnitude. It was an event that, for several reasons, could not be predicted. Above all, it found health institutes structurally unprepared, since they had been previously penalised by reductions in public expenditure, the growing shortage of medical and nursing staff, and the fragmentation of the national system into 21 different regional health systems, which proved very difficult to coordinate.

Each regional health system thus faced the emergency differently, creating a lack of homogeneity in managing those infected. Hospitalisation in Lombardy and Piedmont was between 50% and 60% at the beginning of the pandemic period, and then grew and fluctuated between 70% and 80% in the first half of March 2020, while it decreased in the other regions and finally dropped below 20% at the end of April 2020. While Lombardy opted for hospitalisation, other regions such as Veneto opted for less hospitalisation, preferring home isolation instead.

In all of the regions, however, the following became necessary [3]:

- An increase in the number of beds in the Intensive Care Units (ICU);
- The activation of Special Units of Continuity—USCA (according to legislative decree n. 14 of 9 March 2020, art. 8), with the specific mandate to manage homecare patients who did not require hospitalisation;
- The identification of suitable accommodation facilities and conversion of existing social and healthcare facilities into intermediate facilities for COVID-19 patients.

The management of the emergency, therefore, implied elevated total costs for the admission of the patients affected by COVID-19 and a significant reduction in ordinary admissions, which were continuously delayed and which, in turn, had a further impact on the increase in health expenditure.

The increase in hospitalisations also led to the urgent recruitment of many medical and nursing staff. The data on staff integration ranges for the most affected regions are included in Table 3.

As a litmus test, a particularly critical market issue was that of personal protective equipment (PPE). PPE proved to be essential for the containment of the epidemic outside hospitals and fundamental in managing the epidemic within health facilities, in particular for the protection of health workers [4,5]. Studies in the literature have highlighted the difficulties in supplying these devices in different countries [3,6,7,8] and, in particular, Dai et al. [9] highlighted that from the very beginning of the pandemic spread, there was a clear lack of availability of PPE, while Pecchia et al. [10] analysed the inadequacy of the European regulation for the PPE certification process in an emergency context. For these reasons, to proceed in purchasing these devices, it was necessary everywhere to accelerate the ordinary procedures due to the unavoidable urgency of the necessary purchases, while always maintaining valid efficiency and effectiveness criteria [10]. In turn, this also led—as we know—to repeated judicial checks in order to prevent purchases of substandard devices, or even fraudulent ones [3].

Thus, this extraordinary condition required all the countries of the world, albeit at different times, to adopt flexible extraordinary measures which were constantly evolving and able to bridge the initial gap of general unpreparedness but, above all, of the present health structures [11,12,13]. It was necessary, in fact, to intervene on several levels to be able to simultaneously increase the capacity of reception facilities [14], to increase the number of staff, and to ensure the supply of goods and services required [15], while maintaining adequate transparency with regard to procurement procedures and sources of supply [16,17]. In order to allow these operations, choices were made about spending and reducing ordinary activities [18,19] in order to maintain as many spaces and people as possible during the emergency. In some cases, new production techniques, as additive manufacturing [20] or 3D-printing [21,22,23,24], have been used to address PPE deficiencies.

To enable health structures to cope with the increasing burdens that were being encountered at this point in the administrative and economic spheres, the Italian government adopted a series of emergency measures where they deviated from the general principles for the acquisition of goods and services, which were broadly consolidated.

In essence, crisis measures were taken which envisaged the postponement or reduction of the competitive confrontation (compulsory in Italy from D.Lgs. 50/2016 in an ordinary period), with the consequent risks involved. In order to avoid the development of automatic and generalised exceptional mechanisms and schemes which would have undermined the principles of transparency and competition, these measures were certainly intended to be temporary and sectoral, and to be limited to what was strictly necessary to overcome the emergency. However, they conditioned and affected past institutionalised practices and forced operators, buyers and suppliers [25] to question their practices. An information note published in December 2020 by the World Trade Organization (WTO) recorded an increase of 16% in imported and exported goods compared to 2019 [26].

In this context, the aim of this paper is to show how the emergency caused by an escalation in the COVID-19 pandemic put a strain on the Italian National Health System and forced purchasing centres to deviate from the ordinary general principles dictated by current legislation, reporting the experience of Spedali Civili Hospital in Brescia, one of the largest health care facilities in Lombardy, which was among the most affected areas.

The specific purchase procedures and the rules dictated during the emergency phase will be reported and described with respect to the ordinary practices, as well as the extent to which the two sets of procedures have been integrated. All of these elements will be reflected, taking as a reference period the first phase of COVID-19, limited to the period February–May 2020.

## 2. Materials and Methods

### 2.1. Methods

Considering the current difficulty in finding fully reliable data with a good degree of certainty for many classes of elements useful to provide evidence of analysis, the materials that constitute the field of observation of this work can be defined as follows:

1. The percentage variation of increase in medical/health staff concerning the departments of the entire hospital and in relation to the Complex Operative Unit (U.O.C.), Clinical Engineering, according to Equation (1)
  (1) $$\%\mathrm{variation}=100\times \frac{\mathrm{value}\;\mathrm{at}\;31.05.20-\mathrm{value}\;\mathrm{at}\;31.01.20}{\mathrm{value}\;\mathrm{at}\;31.01.20}$$
2. The rate of increase in ICU beds.
3. The percentage of acquisition and the type of acquisition of specialised biomedical equipment for intensive care and first emergency management intervention.
4. The percentage of tests carried out.
5. The procedures concretely introduced and the fundamental factors of deviation from the ordinary procedure of purchase management.

All the data processed derived from internal sources of the hospital, such as the U.O.C. Planning and Management Control, U.O.C. Management Service, U.O.C. Human Resources and Bed Management Service.

Concerning point 5, it is necessary to begin with a clarification that helps to frame beforehand the response given by Spedali Civili Hospital in Brescia to the complex moment of the emergence of new and unavoidable needs with regard to the acquisition of goods and services.

The procedural steps used, as mentioned, had to take their cue from the national regulatory framework of emergency reference health determined by D.L. n. 18/2020 and in response to legislative decree 50/2016, art. 63, comma 2, letter c) and 163.

According to these frameworks, in addition to the ordinary acquisition channels, namely acquisition through the national and regional central purchasing agency known as Public Information Services (CONSIP), the Regional Company for Innovation and Purchases (ARIA), and the direct purchase procedures by Spedali Civili Hospital, it was possible to acquire goods and services through donations from private individuals or from the National Service of Civil Protection, which, to cope during the period, was commissioned with, among other tasks, the national procurement and local distribution of medical devices (legislative decree n. 9/2020, art. 34 and legislative decree n. 18/2020, art. 5bis).

To obtain an idea of the ‘perturbative’—and potentially overwhelming—impact of the input of products supplied directly by Civil Protection, one can refer to the following table (Table 4).

The volume of products that could not have been subjected to a strict quality control screen is identified quantitatively.

According to D.L. 18/2020, art. 5-bis, paragraph 3, the production, import, and marketing of surgical masks and other PPE were allowed for an emergency period after a preliminary assessment by the National Institute of Health (ISS) and the National Institute for Insurance against Accidents at Work (INAIL), even in the absence of the CE conformity mark and deviating from current provisions. In this widening of the range of acquisition methods, it is useful to point out that the priority of the U.O.C. Clinical Engineering Unit of the Spedali Civili Hospital in Brescia was to maintain, for all intents and purposes, the ‘bar’ of CE conformity. That is, to preserve, in any case, at this particularly delicate and chaotic time, through this fundamental choice, a principle of order with respect to the supply of goods and services indispensable for the departments in charge of the emergency management of COVID-19. In fact, on the same level, it was necessary to work with both absolute timeliness and in precise limitation of the risks, which were not primarily economic ones, but rather those of disruption or even of a feared and nefarious de facto interruption of service.

### 2.2. Data Analysis

The analysis followed a qualitative description and quantitative presentation of the findings using frequency distribution, percentages, and graphs.

Qualitative trend analysis of the data for each topic was used to identify the major issues for each of the main themes.

## 3. Results

In this context, the Spedali Civili Hospital in Brescia welcomed the first positive patient as early as 23 February 2020, coinciding with the first clear signs of the epidemic phase in Italy. In fact, it can be added for contextualisation purposes that the identification in Codogno of what will long be erroneously considered ‘patient zero’ was on 21 February. The declaration by the WHO of the general status of the ‘pandemic’ is dated 11 March 2020. After just over a month, on 2 April 2020, the number of COVID-19 patients rose to, with a more than exponential increase, 2582, subdividable by the personal situation and hospitalisation reported in Table 5.

To cope with the emergency and the increasing number of patients to be accommodated, a race opened up to increase, prepare, and designate adequate facilities for pandemic containment and, as a priority, to protect the healthcare personnel.

**Figure 2:**
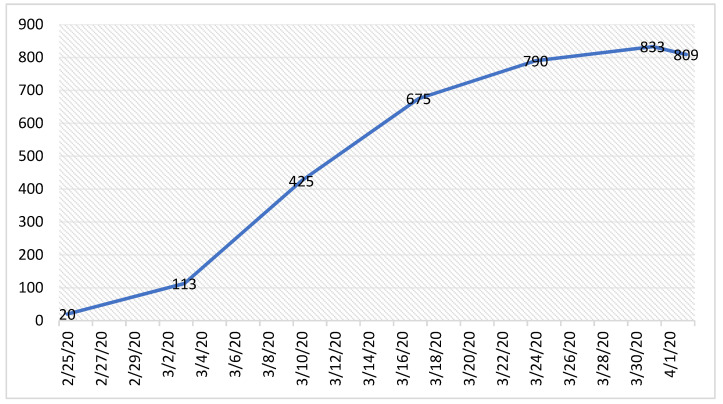
Trend of Spedali Civili Hospital beds from 25 February to 2 April 2020.

It was immediately necessary to expand the number of beds in the ICU departments, even to the detriment of those normally used for ordinary patients (Figure 2).

From the first days of the emergency, a Crisis Unit was established [27] under the direct control of the Administrative Director with technical–administrative functions, which saw the direct involvement of UOC Clinical Engineering. The Crisis Unit answered to the Regional Crisis Unit (UCR), composed of internal and external personnel, which acted as a reference point to offer support and solutions to the problems associated with managing an emergency event, as it had access to multidisciplinary resources.

The first choices that this unit had to carry out immediately were the restructuring of resuscitation 1 to make it a ‘Red’ area, the opening of a new area for 20 beds in infectious diseases, and the construction of an external structure for emergency and pre-hospitalisation admission, constituting the first module of 16 beds prepared for this function.

The next action was the construction of a field hospital located near the emergency room, which was made operational on 27 February. This structure was dedicated to patients with respiratory symptoms or other symptoms related to COVID-19 to avoid congestion in the ordinary ER. The dedicated First Aid area was equipped with a reception for patients who presented themselves, a clinic of triage and examination, radiological equipment, 16 beds for observation of 12 h and/or waiting for swabs, and a waiting area for relatives.

The day after the opening of the field hospital, 32 additional beds were made available in a covered structure with a toilet, to which 8 beds inside a tent were then added.

Furthermore, on 18 March, 45 additional beds were set up in the laundry of the Spedali Civili Hospital in Brescia and 20 beds in the canteen of the Hospital of Montichiari. Equipping a reception area for 20 people at the Emergency Department of the Hospital of Gardone Val Trompia was also arranged.

The overall transformation of the beds allowed a considerable extension of those available and a conversion of the ordinary ones for a final total of 815 beds for COVID patients.

Meanwhile, the staff involved in the transformation reached 4500. As can be seen from the table below (Table 6), the ‘frontline’ roles increased by 3.8%; the health roles, which included doctors, by 2.3%; and technical roles by 5.3%.

Whilst the expansion operations were being carried out, outpatient activity was limited to urgent activities only for reasons of protection and employee availability (Table 7).

Furthermore, all activities considered deferrable, except for emergency surgery, obstetrics, psychiatry, oncology, haematology, dialysis, and serious eating disorders, were suspended (Table 8). Between 15 March and 2 April, these units recorded 840 nonCOVID-19 admissions, of which 60 were strokes and 80 were traumas, while 323 admissions were made from 21 February.

Similarly, the total number of admissions registered in the Spedali Civili Hospital ER during the period February–May 2020 also decreased, albeit with different percentages and with a stronger incidence compared to external needs. The terms are visible in Table 9 and Table 10.

During the first wave of COVID-19, despite the large catchment area of the Spedali Civili Hospital in Brescia and the high number of infections that occurred in the province, together with the increase in the number of COVID patients, a marked decrease in admissions related to pathologies or conditions considered to be ordinary was partly induced and registered.

At this point, we begin to have an ‘exact’ perception of the destabilisation in progress and the enormous efforts that had to be made to meet the pressing needs. The strengthening of structures, increase and relocation of human resources, and strategic reconversion were carried out by several hospital units simultaneously, with a great need for synchronisation and central coordination.

In terms of goods and services, a specific emergency procedure was prepared by the Clinical Engineering Unit and the Procurement UOC to ensure a rapid and efficient response and the application of all the services designated to their acquisition.

The Clinical Engineering Unit was thus identified by the procedure as the operating unit of reference to which to direct the requests for new equipment, which originated as a consequence of the increase in demand for health services and the need to prepare the new departments and services and to maintain a level of assurance of the quality diagnostics and therapeutics offered.

This unit took charge of the necessary requirements, which included:

− The evaluation of the technical characteristics and conformity of the products;
− The assessment of the economic and financial coverage;
− The drafting of the purchase proposal, accompanied by the coding of the equipment;
− The transmission of the documentation to the Procurement UOC.

The Procurement UOC proceeded, in turn, to first verify the possible presence of the requested product in the CONSIP or ARIA Central Purchasing Bodies on the Convention Showcase, in the product catalogue, or online shop (NECA). Given a lack of availability through these channels, and in the absence of availability also through the aggregators, it resorted to purchasing goods and services through a procedure pursuant to Legislative Decree 50/2016, art. 63, paragraph 2, letter c, and art. 163, or through the use of a negotiated procedure without prior publication of a notice, or even through procedures of extreme urgency that allowed for direct entrustment. The demands for the needs of the departments were also met thanks to the acquisition of goods and services through Civil Protection or through private donations (Legislative Decree no. 18/2020).

The Civil Protection officers delivered the devices destined for the Spedali Civili Hospital in Brescia to the hardware warehouse. Once they had carried out the equipment testing, the Clinical Engineering Unit then organised its transfer to and installation in the departments concerned.

The purchase of the equipment and furnishings essential for setting up new beds for the Intensive Care Unit thus equipped with adequate ventilators, beds, and monitors led to a total economic expenditure of about EUR 5.5 million over the period in question.

During the emergency period, donations also multiplied. Therefore, the internal procedure also set itself the goal of guaranteeing the necessity, relevance, safety, and quality of the donated products. It was determined to achieve this through the evaluation of all the proposals made by the Clinical Engineering Unit based on specific criteria such as the real utilisation; the spaces available for the conservation of the assets or their placement; the costs of accession, control, and maintenance; and the technical and regulatory compliance with CE requirements. The value of a condition that violated this latter criterion has already been indicated.

The Clinical Engineering Unit only accepted the donations once compliance with the established criteria had been verified. The size of the volumes at stake, expressed on an economic level, is shown in the table below (Table 11).

Parallel to the sudden increase in requests for assistance, there was an urgent need to equip health personnel with adequate PPE as quickly as possible. Usually purchased en masse through the regional or national central purchasing bodies (ARIA or CONSIP), in the first phase of the emergency, the PPE was instead purchased independently, as the central purchasing bodies were not ready to guarantee sufficient supplies. The first PPE provided by ARIA only arrived in mid-March, when the emergency was almost at its peak.

A similar condition affected services. As the coordinated support of the central purchasing bodies was lacking at the beginning of the pandemic, Spedali Civili Hospital had to purchase the necessary services entirely at its own expense.

The supply of services and PPE to be paid by the Spedali Civili Hospital amounted to a budget item of about EUR 2 million in the financial statements.

Further supplies then arrived through ARIA or, also in this case, through Civil Protection, which in the meantime had created a special fund for the procurement of personal protective equipment and health equipment, which was then distributed to the various hospital units (Table 12).

Further supplies came from donations received from different associations and organisations. Some of the equipment received was found to be not in compliance with CE certifications and, for this reason, in compliance with the internal guidelines undertaken, they were not authorised.

In the period from 24 February 2020 to 31 May 2020, the acquisitions, and consequently the installations and tests carried out by the UOC Clinical Engineering, totaled 1842. Of these, those attributable to the types of equipment most in-demand and fundamental for the epidemic’s management can be summarised as follows (Table 13).

In comparison with the previous year (2019), it can be noted that in the same period of that year, 271 installations and tests of new equipment were carried out. There was, therefore, an increase of as much as 579%.

The state of the allocation of beds for intensive care (ICU) is particularly paradigmatic of how the acquisition efforts made could, in perspective, be aimed at the logic of development induced by the so-called Decreto Rilancio, i.e., decree aimed at relaunching the economy (Legislative Decree no. 34/2020).

In Table 14, the data collected and the projections of the case for the Lombardy Region and the specific Spedali Civili Hospital in Brescia are represented compared to one another. The latter will then be better placed in its provincial context.

The purpose of the elaboration is to compare the two territorial entities to evaluate their mutual progression in some key steps, verify their deviations and hypothesis, and forecast for their commitment to the objectives of the regulatory dictate of the Relaunch Decree. In this sense, the four criteria that will be compared are the number of initial beds, their increase in the peak phase, the current state of the situation, and the progress or not towards the targets set by the decree.

The Relaunch Decree stipulated that the previous allocations of beds in intensive care had to be implemented across the national territory (5179 in the pre-COVID period nationwide), with an additional 3500 places to reach the goal of at least 8679 beds. The decree therefore provided that for each region, this structural increase would determine a reference quota equal to 0.14 beds per thousand inhabitants.

Concerning this parameter, elaborated in Table 11 with some extrapolations of data and as indicated in the notes accompanying the table itself, in April 2020, Spedali Civili Hospital in Brescia produced an increase in beds by 150%, which is more than double the percentage for Lombardy in the same period.

Throughout the whole of the following year, this increase differed slightly from the previous one (152.9% increase, with a difference of 2.9% compared with Lombardy, which was 15.1%), showing factually that the number of beds acquired immediately was sufficient throughout the following year to cover the contingent needs.

The deviation of 88.6 missing beds, required to achieve the forecast objective of 174.6 beds hypothesised by the Relaunch Decree, should therefore not be interpreted as a shortage, but needs to be put into context by comparing it with the number produced at the same time by the other hospital units active in the Province of Brescia. The figure of 6.8 beds (for the peak period of April 2020) or 6.9 (for September 2021) thus maintains, in this perspective, a relevance equivalent to that of the regional parameters of increase considered above (13.9 and 15.2 beds). It can thus be concluded that Spedali Civili Hospital in Brescia has covered—practically since the beginning of the emergency—not only half of the Province of Brescia’s bed requirements, but above all, an adequate quantity for the hospitalisation needs of the most acute states of the crisis that would have followed.

## 4. Discussion and Conclusions

The analysis carried out on Spedali Civili Hospital in Brescia and the evidence produced shows how, in the three crucial months for the management of the emergency, when the reception and intervention capacity capable of supporting the crisis was achieved, not only was a new service established but also, more importantly, a ‘new operating regime’ was established. From an administrative and managerial point of view, the strong acceleration required by the needs of the departments did not merely make the organisational structure of the hospital difficult but forced it to reassess its purchasing procedures and to revise them, as has also been noted in the literature for other countries [3,7,14]. Several studies have highlighted and analysed the fundamental criticalities found [28], in particular those resulting from the significant supply difficulties of certain commodity categories [16,29]. Okeagu et al. [16] discussed supply chain management, governance and financing, emergency protocols, including emergency procurement and supply chain, supply chain gaps and how to address them, and the importance of communication in times of crisis. Handfield et al. [29] reviewed existing approaches to managing the strategic national stockpile and contingency sourcing and pandemic plans in the United States, and then they mapped out the supply chain’s responses to the national contingency planning phases. Bhaskar et al. [30] highlighted the fragility of the supply chain during COVID-19 and suggested the creation and implementation of a framework with the maximum interaction between public and private companies in order to no longer witness a failure in this system. In other studies, the supply of PPE [31], ventilators [32] and equipment for the provision of intensive care beds [30], and also the pharmaceutical supply chain [33,34,35], was revealed to be very critical [36,37]. All these criticalities in low-income countries have also been amplified, as we can see in the works of Tran et al. [38], Aljadeed et al. [39] and Karunathilake [13]. These works have shown that the common effort will have to be increasingly directed to the improvement of the technology able to predict the potential deficiencies as well as to the implementation of procedures [30] and proactive fiscal strategies to meet requirements [29]. These critical elements have also been detected within Italian health structures and have allowed us to conduct a specific analysis based on them in a more limited context.

From the presented data related to Spedali Civili Hospital, it is possible to observe many unpredictably ‘contradictory’ movements recorded in the first phase of the pandemic. While admissions to the emergency room decreased (Table 9 and Table 10), the hospitalised patients presenting the same new symptomatology prompted the emergency retrieval of many biomedical devices (see Table 13). The reduction in outpatient activities (Table 7) was counterbalanced by an exponential increase in workload for both health and technical and administrative personnel, with the constant perception of not being able to withstand the impact of the increased demand. The introduction of new dedicated employees (Table 6) also contributed to ensuring that the workforce of the entire institute focused on the management of the health emergency, but this occurred at the expense of ordinary health management.

Once the emergency phase was overcome, the creation of additional intensive care beds did not imply the obligation to maintain all of the additional beds as fully and effectively operational unless otherwise indicated by the region; however, their creation at the same time presupposes the offer of their future availability at a time of need.

If at first compatible with the acute phase, the structures and subjects involved were unprepared, at a later stage. It was possible to develop a perspective of structural evolution that can adapt to the needs and at the same time safeguard the minimum standards for the effective use of the acquired equipment and the services provided.

In particular, transparency and security were maintained, quality and control of expenditure were reconciled, and the quantitative presence of the instruments as well as their correct functioning were guaranteed. The actions deviating from the ordinary procedures led to a streamlined and revised operational process, for which the key components were highlighted in the strategically distributed evaluation steps and the communication nodes of process synthesis.

Thus, according to this Italian experience and the literature, the importance of identifying optimal strategies for the planning of spending budgets available, the development of technologies suitable for the management of any health emergencies, the development of digitalization process [40,41], and the improvement of the supply chain in terms of availability of goods and their manage utilisation was evident [33,36,37].

Required for the future management of emergency events of similar magnitude will be the provincial control of the potential capacity of intensive care beds to activate an adequate number of locations in case of need.

In addition, the study and drafting of procedures that are effective but at the same time also simplified and adaptable to different needs will become increasingly fundamental. In this sense, a cultural investment will need to be prepared and supported: first for the training of personnel, in a logic of work among internal intersectoral networks, to build the capabilities required to guide the processes and to keep them constantly monitored on a qualitative–procedural level, and to gain the appropriate skills for the selection of purchases in calls for tender. However, this cultural investment will also require achieving a strategic reformulation of the general objectives to be pursued, both in an anticipatory and preventive perspective (in particular, thinking of the PNRR perspective).

In the specific case of the Clinical Engineering Unit, in addition to the development and internal review of technical and administrative functions and procedures, it must be recognised that it played a decisive role in the implementation of the regional and national HTA (Health Technologies Assessment) network, which through sharing with the other hospital units concerned, will be able to guarantee an appropriate verification of the purchases of the necessary technologies. This will be possible with due adjustments to the current emergency condition [42].

In conclusion, starting from observations of the great upheaval that the pandemic phenomenon has brought on the level of organisation in health services of the Spedali Civili Hospital in Brescia in particular, this study was the first opportunity to collect data in a structured way to compare the quantitative and procedural differences between the pre-COVID period and the first wave of the COVID period, which, in particular, put a strain on the corporate structure. In addition, it defined a ‘new’ procedure that was determined thanks to the impact of the emergency on ordinary practices and thus identified a procedural form capable of flexibility in case of an emergency that can be integrated into a logic system.

It is important to underline that this study has some limitations. The procedural model that has been formed is presented as a working hypothesis that will have to be further tested at an operational level. The relevant data will then be compared to the first phase of the pandemic; it could be interesting to implement them further and to compare the second wave of the pandemic to explore differences or analogies between the two periods. It is expected that the experience of the first phase may have led to the knowledge and implementation of some working methods which were useful in the second phase and which meant that the hospital and its personnel were not untrained. It is clear that understanding the direct and indirect effects of the COVID-19 pandemic will be important when preparing for future pandemics. The expectation is that all countries will be better prepared for pandemics in the future.

## Figures and Tables

**Table 1:** Total COVID-19-positive cases on 31 May 2020 for the four regions most affected in terms of deaths and for which it was possible to derive all of the data in relation to the first wave of COVID-19.

	Lombardy	Emilia-Romagna	Piedmont	Veneto
24 February 2020	172	18	33	3
2 March 2020	1254	335	273	51
9 March 2020	5469	1386	744	350
16 March 2020	14,649	3522	2473	1516
23 March 2020	28,761	8535	5505	4861
30 March 2020	42,161	13,531	8724	8712
6 April 2020	51,534	17,556	11,588	12,924
13 April 2020	60,314	20,440	14,251	17,134
20 April 2020	66,971	22,867	16,127	21,349
27 April 2020	73,479	24,662	17,579	25,098
4 May 2020	78,105	26,175	18,373	27,622
11 May 2020	81,871	26,876	18,741	28,776
18 May 2020	85,019	27,267	18,950	29,619
25 May 2020	87,258	27,587	19,097	30,228
30 May 2020	88,968	27,790	19,152	30,637

**Table 2:** Geographical areas with the highest percentages of deaths due to COVID-19. The percentage is calculated in relation to the total number of deceased in Italy (33,340).

	Number of Deaths	Percentage of Deaths
Lombardy	16,079	48.23%
Emilia-Romagna	4107	12.31%
Piedmont	3858	11.57%
Veneto	1916	5.74%
Other regions	7380	22.13%

**Table 3:** Percentage changes in employee integration in May 2020.

Regions	Doctors in Service at 31 December 2018	Doctors Hired for COVID-19 Emergency	Change %	Nurses on Duty at 31 December 2018	Hired Nurses for the COVID-19 Emergency	Variation %
Lombardy	15,370	589	+3.8%	36,688	1016	+2.6%
Emilia-Romagna	8948	421	+4.7%	25,626	1032	+4%
Piedmont	8883	269	+3.0%	21,834	692	+3.2%
Veneto	8266	215	+2.6%	24,665	573	+2.3%

**Table 4:** Consumable and non-consumable products distributed by Civil Protection.

Products	Lombardy	Emilia-Romagna	Piedmont	Veneto
Consumables	16,264,896	10,975,969	8,936,845	9,519,916
Non-Consumables	27,200	17,320	16,205	5403
% consumable compared to national	18.37%	12.40%	10.09%	10.75%
% not consumable compared to national	23.24%	14.73%	13.79%	4.60%

**Table 5:** COVID-19 patients at the Spedali Civili Hospital in Brescia as of 2 April, 2020, split by patients in home isolation and those hospitalised. Hospitalised patients may be further divided into discharged, deceased, transferred, and still-hospitalised patients.

Patients Classification	Number of Patients	Percentage of Patients
Discharged at home	580	23%
Discharged	552	21%
Deceased	403	16%
Transferred	238	9%
Still hospitalised	809	31%

**Table 6:** Recruitment of staff between February and May 2020.

Staff	Present at 31 January 2020	Present at 31 May 2020	Increment
Health role (doctors)	4372 (950)	4543 (972)	+3.9% (+2.3%)
Professional role	4	5	+25%
Technical role	1599	1689	+5.6%
Administrative role	571	574	+0.5%

**Table 7:** Outpatient performance reduction indices.

Hospitals	Number of Outpatient Services—Other Services February–May 2019	Number of Outpatient Services—Other Services February–May 2020	Percentage Difference
Spedali Civili Hospital in Brescia	1,030,865	669,317	−35.07%
Hospital of Gardone V.T.	125,388	71,791	−42.74%
Hospital of Montichiari	126,587	68,246	−46.09%
Umberto I Children’s Hospital	78,833	37,580	−52.33
Total	1,361,673	846,934	−37.80%

**Table 8:** In-patient surgeries performed.

Hospitals	Number of Interventions February–May 2019	Number of Interventions February–May 2020	Percentage Difference
Spedali Civili Hospital in Brescia	7778	4436	−42.97%
Umberto I Children’s Hospital	1104	608	−44.93%
Hospital of di Montichiari	550	248	−54.91
Hospital of Gardone V.T.	518	172	−66.80%
Total	9950	5464	−45.09%

**Table 9:** Admissions to the ER.

Hospitals	Number of Admissions February–May 2019	Number of Admissions February–May 2020	Percentage Difference
Hospital of Montichiari	1108	1158	4.51%
Hospital of Gardone V. T.	812	803	−1.11%
Umberto I Children’s Hospital	892	691	−22.53%
Spedali Civili Hospital in Brescia	7128	6548	−8.14%
Total	9940	9200	−7.44%

**Table 10:** Admission to the ER for outpatient patients.

Hospitals	Number of Admissions February–May 2019	Number of Admissions February–May 2020	Percentage Difference
Hospital of Montichiari	4900	2696	−44.98%
Hospital of Gardone V. T.	4922	2573	−47.72%
Umberto I Children’s Hospital	13,100	5739	−56.19%
Spedali Civili Hospital in Brescia	20,941	12,191	−41.78%
Total	43,863	23,199	−47.11%

**Table 11:** Amount of equipment donations received by Spedali Civili Hospital in Brescia between March and May 2020.

Donor	Value of Equipment Donated (Expressed in EUR)
Civil Protection	EUR 77,550.00
Other entities	EUR 2,226,858.24

**Table 12:** PPE delivered to the Spedali Civili Hospital in Brescia from 28 February 2020 to 31 May 2020.

PPE	ARIA	Donations	Purchases
Single-use shoe covers	153,880	32,200	42,000
Protective visors	76,180	3115	90
Disposable protective coveralls with hood and elastic cuffs and ankles (Cat 1–Cat 3)	23,072	7845	3200
Splash goggles	10,040	1237	-
Disposable surgical caps	-	1000	600

**Table 13:** Number of installations and tests carried out between February and May 2020.

Equipment	Installations and Tests				
	February	March	April	May	Total
Surgical–medical aspirator	0	41	4	7	52
Monitoring station	0	2	0	1	3
Defibrillator	0	9	6	2	17
Chest compression system	0	1	24	0	25
Echotomography	1	4	8	3	16
Portable ultrasound scan	1	3	5	3	12
Electrocardiograph	0	2	11	0	13
Blood gas analyser	0	2	1	0	3
Electro-controlled bed for intensive care	0	28	4	0	32
Electric hospital bed	0	18	34	24	76
Monitor	0	114	28	9	151
Enteral feeding pump	0	20	13	3	36
Syringe pump	0	157	21	0	178
Infusion pump	0	1	106	0	107
Continuous positive pressure equipment	0	9	26	10	45
Pulse oximetry	12	155	192	9	368
Ultrasound probe	3	19	29	15	66
Pump support	0	0	13	0	13
Syringe pump support	0	12	18	0	30
Thermometer	0	37	22	1	60
Tympanic thermometer	0	0	34	0	34
Humidifier	2	11	21	2	36
Ventilator for extra-hospital use	0	0	38	10	48
Ventilator for hospital use	0	88	30	2	120
Transportable emergency ventilator	0	0	5	5	10
Video laryngoscope	0	3	2	0	5
Total	19	736	695	106	1556

**Table 14:** Intensive care beds in relation to the acquisitions made and in perspective.

Hospitals	(a) Beds in ICU Pre-COVID	(b) Beds in ICU—April 2020 Peak	(c) Current Beds in ICU— 8 September 2021	(a) Beds Pre-COVID Expressed/100,000 Residents	(b) Beds—April 2020 Peak Expressed/100,000 Residents	(c) Current Beds—8 September 2021 Expressed/100,000 Residents	Forecast Beds Based on DR Expressed/100,000 Residents	Current Deviation from the Forecast
Lombardy	861	1400 Increase percentage = 62.6%	1530 Increase percentage = 77.7%	8.6	13.9 *	15.2	146.0	84.0
Spedali Civili Hospital in Brescia	34	85 Increase percentage = 150%	86 Increase percentage = 152.9%	2.7	6.8 **	6.9	174.6 ***	−88.6 ***

Legend: ICU = Intensive Care Unit; DR = Relaunch Decree. * The data were estimated based on the size of the Lombardy population on 1 January 2020 (source: ISTAT): 10,060,574 units. ** The data were estimated based on the size of populations in the Brescia province on 1 January 2020 (source: ISTAT): 1,246,983 units. *** The forecast objective of 174.6 beds for the province of Brescia was obtained by considering the parameters set by the DR based on the population in the province of Brescia.

## Data Availability

All data analysed during this study are included in this published article.
